# The ontogenic gonadal transcriptomes provide insights into sex change in the ricefield eel *Monopterus albus*

**DOI:** 10.1186/s40850-022-00155-4

**Published:** 2022-11-23

**Authors:** Miao Fan, Wei Yang, Weimin Zhang, Lihong Zhang

**Affiliations:** 1 Institute of Aquatic Economic Animals and Guangdong Province Key Laboratory for Aquatic Economic Animals, School of Life Sciences, Sun Yat-Sen University, Guangzhou, People’s Republic of China; 2 Present address: Institute of Biomedical Engineering, Shenzhen People’s Hospital (The Second Clinical Medical College, Jinan University; The First Affiliated Hospital, Southern University of Science and Technology), Shenzhen, 518020 Guangdong People’s Republic of China; 3 Biology Department, School of Life Sciences, Sun Yat-Sen University, Guangzhou, People’s Republic of China

**Keywords:** Ricefield eel *Monopterus albu*s, Gonad, Sex change, Biopsy, Transcriptome

## Abstract

**Background:**

The ricefield eel is a freshwater protogynous hermaphrodite fish and has become an important aquaculture species in China. The sex change of ricefield eel is impeding its aquaculture practice, particularly the large-scale artificial breeding. Many studies including transcriptomes of mixed gonadal samples from different individuals have been aimed to elucidate mechanisms underlying the sex change. However, the key physiological factors involved in the initiation of sex change remain to be identified.

**Results:**

The present study performed transcriptomic analysis on gonadal samples of different sexual stages obtained through biopsy from the same fish undergoing sex change. A total of 539,764,816 high-quality reads were generated from twelve cDNA libraries of gonadal tissues at female (F), early intersexual (EI), mid-intersexual (MI), and late intersexual (LI) stages of three individual sex-changing fish. Pairwise comparisons between EI and F, MI and EI, and LI and MI identified 886, 319, and 10,767 differentially expressed genes (DEGs), respectively. Realtime quantitative PCR analysis of 12 representative DEGs showed similar expression profiles to those inferred from transcriptome data, suggesting the reliability of RNA-seq data for gene expression analysis. The expression of *apoeb*, *csl2*, and *enpp2* was dramatically increased and peaked at EI while that of *cyp19a1a*, *wnt4a*, *fgf16*, and *foxl2a* significantly downregulated from F to EI and remained at very low levels during subsequent development until LI, which suggests that *apoeb*, *csl2*, *enpp2*, *cyp19a1a*, *wnt4a*, *fgf16*, and *foxl2a* may be closely associated with the initiation of sex change of ricefield eels.

**Conclusions:**

Collectively, results of the present study confirmed that the down-regulation of female-related genes, such as *cyp19a1a*, *wnt4a*, *fgf16*, and *foxl2a*, is important for the sex change of ricefield eels. More importantly, some novel genes, including *apoeb*, *csl2*, and *enpp2*, were shown to be expressed with peak values at EI, which are potentially involved in the initiation of sex change. The present transcriptomic data may provide an important research resource for further unraveling the mechanisms underlying the sex change and testicular development in ricefield eels as well as other teleosts.

**Supplementary Information:**

The online version contains supplementary material available at 10.1186/s40850-022-00155-4.

## Background

Most vertebrates are gonochoristic, in which individual organisms contain only male or female sex organs throughout their lifetime [1, 2]. In contrast, hermaphrodites in which individual organisms contain both male and female sex organs, are observed in about 2% of all extant teleost species scattering across more than 20 taxonomic families in 9 orders, which can be categorized into sequential and synchronous modes [3]. The sequential hermaphroditism can change their primary sexual status and takes on three forms including protogynous (female-to-male), protandrous (male-to-female), and serial bidirectional sex change [4]. How natural sex change may initiate and progress remains to be elucidated [5]. Natural sex change involves coordinated transformations across multiple biological systems, including behavioural, anatomical, neuroendocrine, and molecular axes, but the molecular basis underlying this amazing transformation remains poorly understood [6].

Ricefield eel *Monopterus albus*, a member of the order Synbranchiformes, is a protogynous hermaphrodite [7] and widely distributes throughout Asia [8]. It is one of the favorite food fish and emerges as an important freshwater cultured species in China with an annual production of more than 300,000 metric tons. The ricefield eel fries for aquaculture are mainly obtained through catching from wild environments and half artificial reproduction [9]. The natural sex change severely affects the sex ratio and thus decreases the productivity of ricefield eel broodstock. The elucidation of the mechanisms underlying the sex change will aid in developing techniques for controlling sex change of ricefield eels.

Previous studies have shown that the treatment with gonadotropin-releasing hormone analogue of Salmon (sGnRH-A) induced the precocious sex change of ricefield eels from females to males and promote the maturation of males [10]. Recently, many sex-related genes have been revealed to be associated with sex change of ricefield eels, including *cyp19a1a* [11], *dmrt1* [12], *foxl2* [13], *gsdf* [14], *17β-hsd12*, *dmc1*, *foxn5*, *gal3*, *maats1*, *spag6*, *spef2*, and *zpsbp3* [15]. In addition, epigenetic modifications of *cyp19a1a* gene [16] and gonadal miRNAs [17] have also been shown to be involved in the sex change of ricefield eels. However, the mechanisms triggering the sex change remain unclear, and the effective ways to manipulate sex change of ricefield eels remain to be developed.

The process of sex change of ricefield eels from females to males could be divided into two phases, namely the initiation of sex change and the subsequent gonadal development towards the testis. The mechanisms that initiate sex change should be activated at the very early stage of the sex transformation process. Thus, obtaining gonadal samples at the early stages of sex change is key to explore the mechanisms underlying the gonadal sex change in ricefield eels. Because the initiation of gonadal sex change was asynchronous and shown to differ in the age, bodyweight, and body length among individual ricefield eels [18], the pooled gonadal sample from different individuals could be a mixture of gonads at different developmental states during sex change and this may hinder the identification of key genes responsible for the process of gonadal transformation. By following the biopsy technique developed previously [19], our present study traced the gonadal transformation process of female ricefield eels, and obtained the gonadal samples at the female, early intersexual, mid-intersexual, and late intersexual stages from the same individual ricefield eel. Ontogenic transcriptomic analysis of these gonadal samples revealed the shutdown expression of *cyp19a1a* (encoding the gonadal aromatase in teleosts) and decreased expression of *foxl2a* in gonads at the early intersexual stage. Moreover, some novel genes such as *apoeb* and *enpp2* were shown to be expressed with peak values at the early intersexual stage, which may be potentially involved in the initiation of gonadal sex change. Results of present study may aid in further understanding the underlying mechanisms of the gonadal sex change in ricefield eels as well as other protogynous sex changing teleosts.

## Results

### Ontogenic gonadal histology of sex changing ricefield eels

**Fig. 1:**
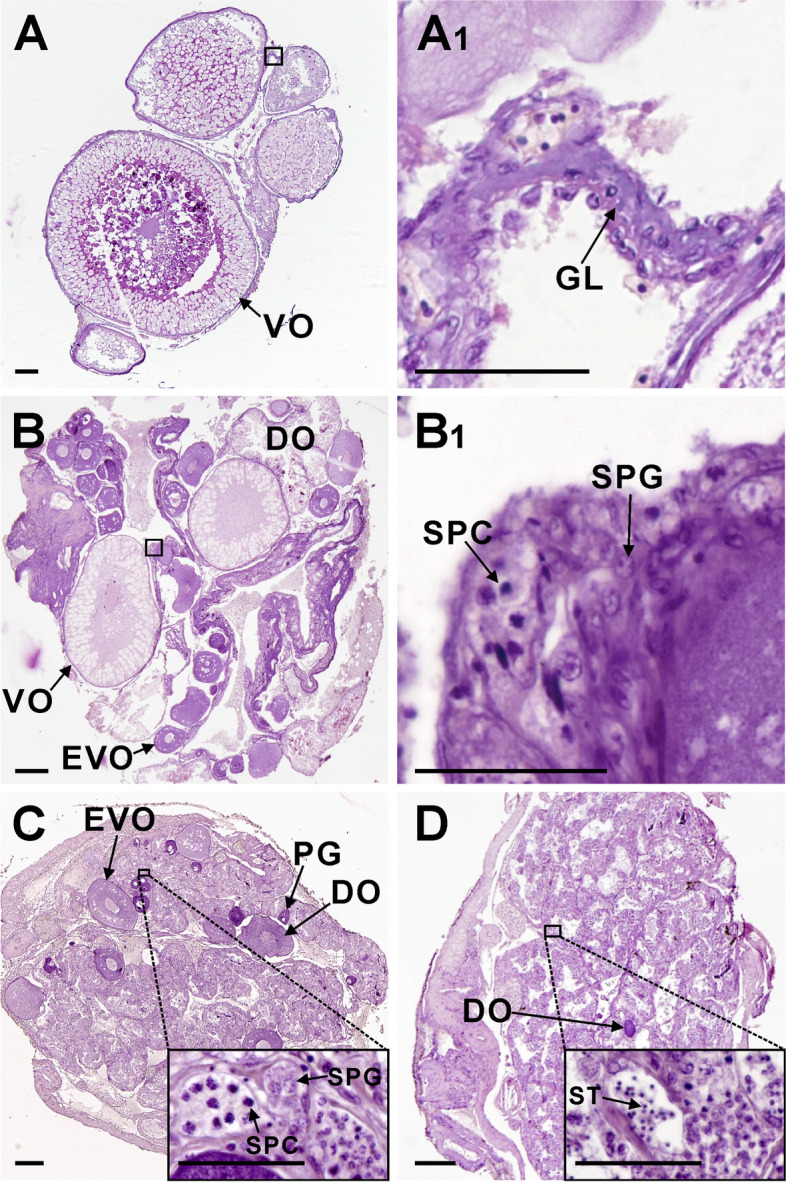
The histology of gonadal samples obtained from a representative sex changing ricefield eel (S7) through biopsy. **A**, the gonad at female stage. **B**, the gonad at the early intersexual stage. **C**, the gonad at the mid-intersexual stage. **D**, the gonad at the late intersexual stage. A1 and B1 are higher magnification of the black-line boxed areas in A and B, respectively. The insets within C and D are higher magnification of the corresponding boxed areas. F, female; EI, early intersex; MI, mid-intersex; LI, late intersex; GL, gonadal lamellae; PG, primary growth stage oocyte; EVO, early vitellogenic oocyte; VO, vitellogenic oocyte; DO, degraded oocyte; SPG, spermatogonia; SPC, spermatocyte; ST, spermatid. Scale bar = 100 µm

Among the thirty-six female ricefield eels biopsied, three (designated as S5, S7, and R10, respectively) were shown to undergo sexual changes with similar developmental pace from female to late intersexual stages during the experiment. Gonadal samples at female (F), early intersexual (EI), mid-intersexual (MI), and late intersexual (LI) stages were obtained from each of the three ricefield eels, respectively. The female gonad contained thin gonadal lamellae with oocytes at the vitellogenic stage (Fig. 1 A and A1). The early intersexual gonad contained thickening gonadal lamellae with oocytes at early growth stages and a few spermatogenic cysts containing SPG or SPC (Fig. 1 B and B1). The mid-intersexual gonad contained a mass of male germ cells including spermatogonia and spermatocytes, and a few oocytes at early growth stages or under degradation (Fig. 1C). The late intersexual gonad contained numerous spermatogenic tubules and a very few degrading oocytes (Fig. 1D). The quality of total RNA isolated from gonadal samples was shown to be suitable for transcriptome sequencing by agarose gel electrophoresis (Additional file 1: Figure S1) and an Agilent 2100 Bioanalyzer (Agilent 2100, Agilent Technologies, USA; the minimum value of RIN after RNA extraction is 7.7).

### Quality control parameters of RNA-Seq data

Twelve cDNA libraries were constructed with the total RNA extracted from gonadal tissues of ricefield eels at stages of female (*n* = 3), early intersex (*n* = 3), mid-intersex (*n* = 3), and late intersex (*n* = 3), respectively. The cDNA libraries were sequenced on an Illumina Hiseq platform and generated a total of 541,302,380 raw reads, which generated 539,764,816 high-quality reads and 80.4 Gb high-quality bases after the removal of adapter sequences and low-quality reads, with more than 37,250,062 high-quality reads and 5.5 Gb high-quality bases from each cDNA library. The percentage of bases with a Phred value of at least 20 (Q20) was higher than 96.78%, and the percentage of bases with a Phred value of at least 30 (Q30) was higher than 91.53% (Supplementary Dataset File: Table S1). Guanine Cytosine (GC) contents were around 51% for all the libraries (Supplementary Dataset File: Table S1). The percentages of high-quality reads that were mapped to the ribosome database ranged from 0.14% to 2.92% (Supplementary Dataset File: Table S2), which were removed. The left high-quality reads were annotated against the ricefield eel (*Monopterus albus*) reference genome (https://www.ncbi.nlm.nih.gov/genome/24053?genome_assembly_id=302095). An average percentage of exon mapped reads higher than 84.6% was obtained for all the libraires (Supplementary Dataset File: Table S3), which suggests that the reference genome was well-annotated. A total of 23,558 genes were identified in the transcriptome data, of which 20,941 were refer genes and 2,617 were novel genes (Supplementary Dataset File: Table S4 and Table S5).

### Differentially expressed genes (DEGs) and enrichment analysis in gonads during sexual change

**Fig. 2:**
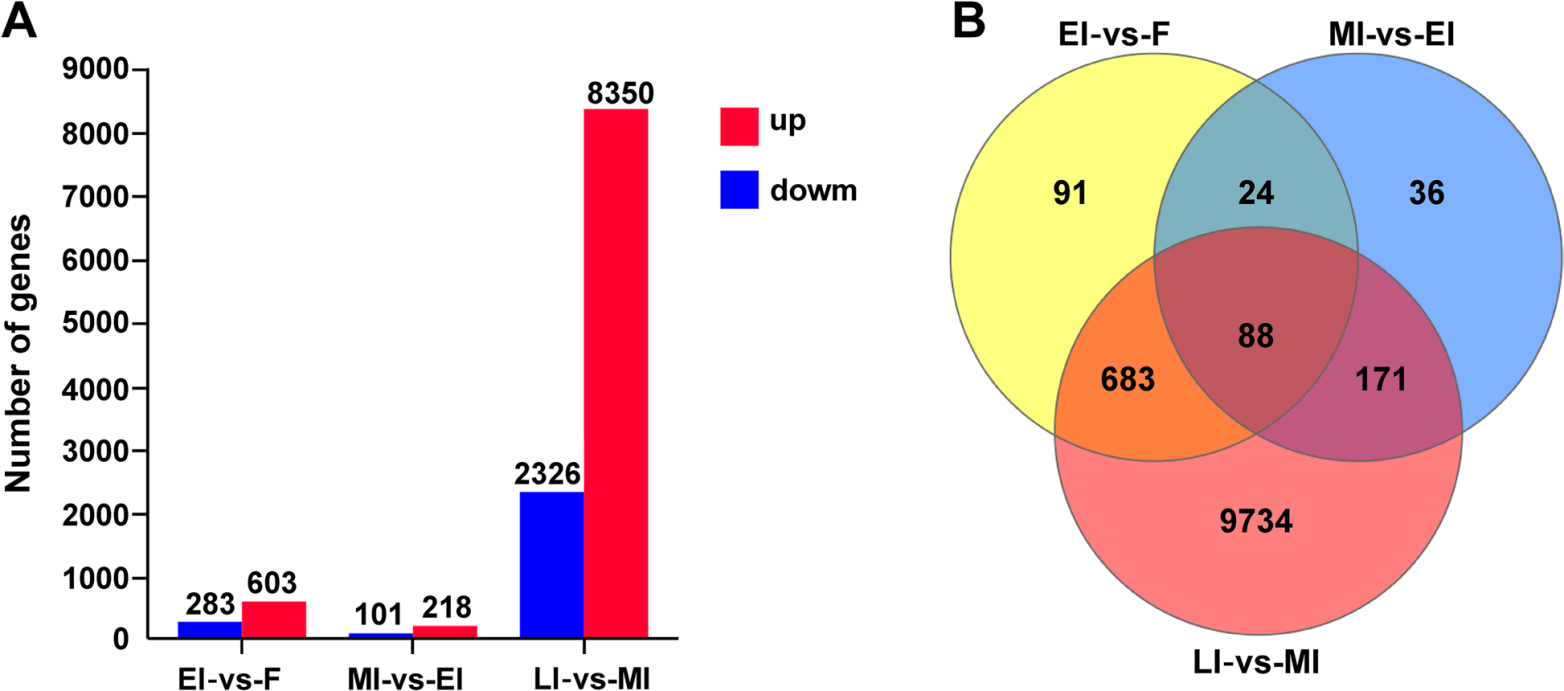
The histogram and venn diagram of the number of DEGs in transcriptome data of gonads of different sexual stages. **A,** the histogram shows the number of up-regulated and down-regulated DEGs for the different pairwise comparisons. Y-axis shows the number of DEGs, and X-axis shows the comparison groups. up: up-regulated genes; down: down-regulated genes. **B,** the venn diagram showing the number of DEGs specific or common to the comparisons. F: female; EI: early intersex; MI: mid-intersex; LI: late intersex

Analysis of RNA-seq data identified 886, 319 and 10,676 DEGs from pairwise comparisons between F and EI (EI vs. F), EI and MI (MI vs. EI), MI and LI (LI vs. MI), respectively (Fig. 2). Up-regulated and down-regulated genes were 603 and 283 for EI vs. F, 218 and 101 for MI vs. EI, and 8,350 and 2, 326 for LI vs. MI, respectively (Fig. 2A). There were 9,734 DEGs only identified between MI and LI (Fig. 2B), which represents about 89.87% of total DEGs and suggests that dramatic developmental changes occurred in gonads from stage MI to LI.

**Fig. 3:**
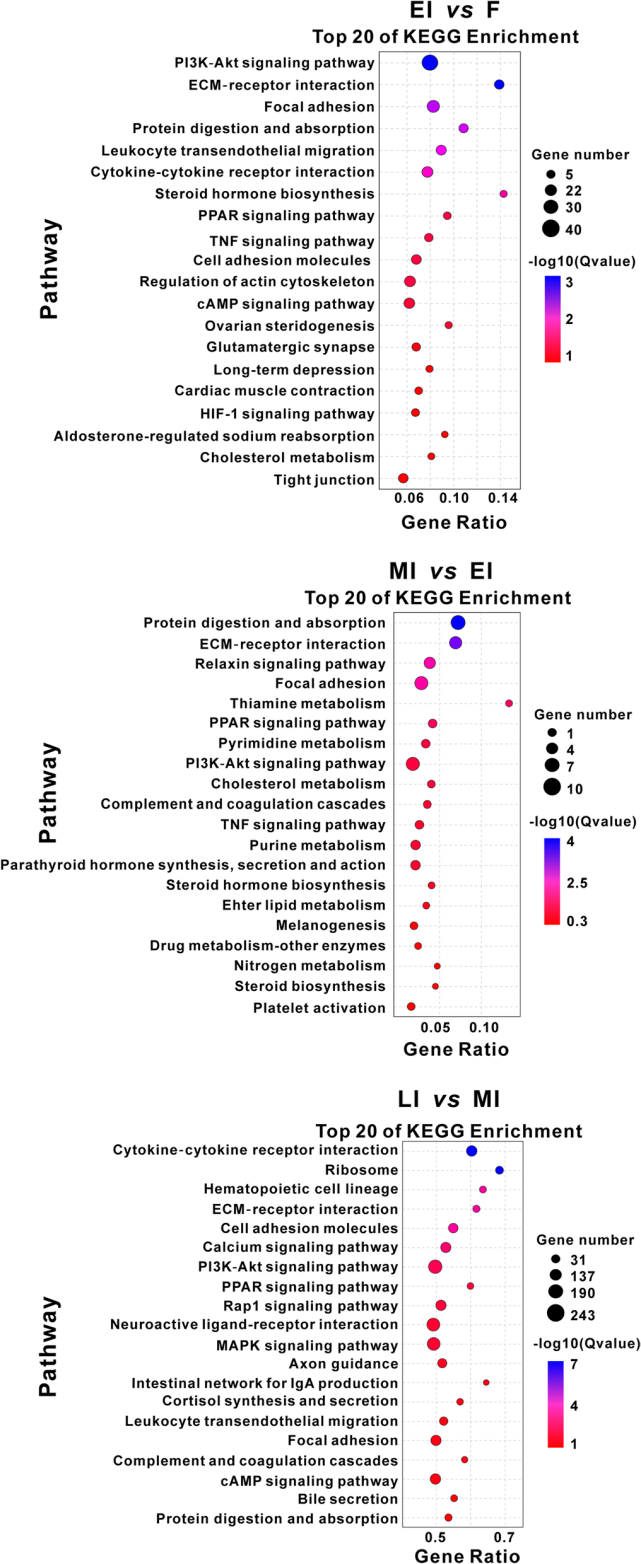
The top 20 of KEGG pathways of enrichment in comparisons between gonads at different sexual stages. The gene ratio is the number of DEGs as a percentage of the total number of genes in a KEGG pathway [20]. The color of the plot represents the *q-*value (negative log10 transformed) of the enriched pathway, and the twenty pathways with minimal *q-*values were defined as the top 20 pathways. F: female; EI: early intersex; MI: mid-intersex; LI: late intersex

Gene ontology analysis was performed on DEGs for EI vs. F, MI vs. EI, and LI vs. MI, respectively, which showed similar enrichments for all the comparisons (Additional file 1: Figure S2; Supplementary Dataset File: Table S6, S7 and S8). KEGG pathway enrichment analysis showed that PI3K-Akt signaling pathway (ko04151), protein digestion and absorption (ko04974), ECM-receptor interaction (ko04512), cholesterol metabolism (ko04979), and steroid hormone biosynthesis (ko00140) were frequently observed during gonadal transition from the ovary to the mid-intersexual ovotestis. DEGs identified during gonadal transition from the mid- to late intersexual ovotestes were enriched in cytokine-cytokine receptor interaction (ko04060), ribosome (ko03010), hematopoietic cell lineage(ko04640), ECM-receptor interaction (ko04512), and cell adhesion molecules (ko04514) (Fig. 3; Supplementary Dataset File: Table S9, S10 and S11).

### The expression of DEGs for EI vs. F during sex change from female to late intersexual stages

**Fig. 4:**
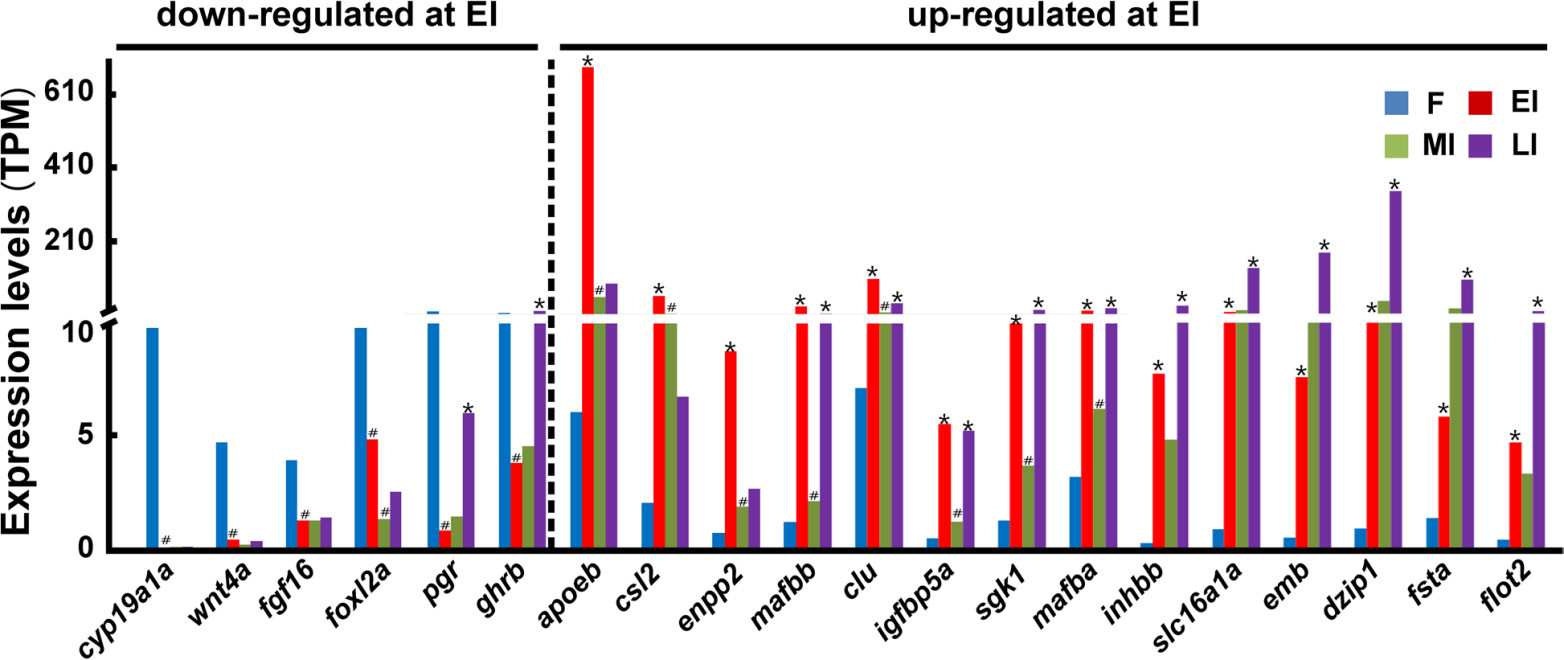
The expression of representative DEGs for EI vs. F in gonads of ricefield eels during sexual change from female to late intersexual stages. Y-axis shows TPM values of genes inferred from the transcriptome data. X-axis shows symbols of genes, and *Entrez* (NCBI) IDs are 109963333 (*cyp19a1a*), 109,971,989 (*wnt4a*), 109,971,151 (*fgf16*), 109,951,921 (*foxl2a*), 109,968,475 (*pgr*), 109,973,650 (*ghrb*), 109,954,649 (*apoeb*), 109,967,173 (*csl2*), 109,973,198 (*enpp2*), 109,955,915 (*mafbb*), 109,962,336 (*clu*), 109,968,774 *(igfbp5a*), 109,956,374 (*sgk1*), 109,964,672 (*mafba*), 109,963,795 (*inhbb*), 109,951,032 (*slc16a1a*), 109,959,091 (*emb*), 109,951,177 (*dzip1*), 109,956,679 (*fsta*), and 109,973,748 (*flot2*). F: female; EI: early intersex; MI: mid-intersex; LI: late intersex. *up-regulated significantly as compared to that at the previous stage; ^#^down-regulated significantly as compared to that at the previous stage

In order to search for genes potentially involved in the initiation of sex change, the expression DEGs for EI vs. F during sex change was further examined. The representative down-regulated DEGs include *cyp19a1a* (cytochrome P450 aromatase), *wnt4a* (wingless-type MMTV integration site family, member 4a), *fgf16* (fibroblast growth factor 16), *foxl2a* (forkhead box L2a), *pgr* (progesterone receptor), and *ghrb* (growth hormone receptor b). The expression of *cyp19a1a* mRNA was decreased by 100% at EI, and remained at very low levels from MI to LI with TPM values less than 0.1. The expression of *wnt4a* mRNA was decreased by 91% at EI, and then remained at similarly low levels thereafter with TPM values less than 0.4. The expression of *fgf16* mRNA was decreased by 65% at EI, and then remained at similarly low levels thereafter with TPM values less than 1.3. The expression of *foxl2a* mRNA was decreased from F to MI by about 88%, and then slightly increased at LI with a TPM value less than 2.5. The expression of *pgr* and *ghrb* mRNA was decreased at EI by 96% and 73%, respectively, and then increased from MI to LI (Fig. 4; Supplementary Dataset File: Table S12).

The 603 up-regulated DEGs for EI vs. F (Supplementary Dataset File: Table S13) showed different expression patterns during gonadal sex change. Some DEGs, including *apoeb* (apolipoprotein Eb), *csl2* (L-rhamnose-binding lectin SML-like), and *enpp2* (ectonucleotide pyrophosphatase/phosphodiesterase 2), were upregulated at EI by about 114-, 30-, and 14.5-fold respectively as compared to that at F, and then their expression decreased significantly thereafter. Some DEGs, including *mafbb* (v-maf avian musculoaponeurotic fibrosarcoma oncogene homolog Bb), *clu* (clusterin)**,** *igfbp5a* (insulin-like growth factor binding protein 5a), *sgk1*(serum/glucocorticoid regulated kinase 1), and *mafba* (v-maf avian musculoaponeurotic fibrosarcoma oncogene homolog Ba), were upregulated at EI by about 28-, 15-,13-, 8-, and 7-fold respectively as compared to that at F, then their expression decreased significantly at MI but increased again at LI to a similar level as that at EI. Other DEGs, including *inhbb* (inhibin subunit beta B), *slc16a1a* (monocarboxylate transporter 1), *emb* (embigin), *dzip1*(zinc finger protein Dzip1-like), *fsta* (follistatin a), and *flot2* (flotillin-2), were increased at EI by about 31-,18-, 15-, 13-, 12-, and 4-fold, respectively as compared to that at F, then their expression remained at a similar level at MI but increased significantly at LI (Fig. 4).

### The expression of genes encoding receptors for gonadotropin-releasing hormones, gonadotropins, and sex steroids in gonads during sex change

**Fig. 5:**
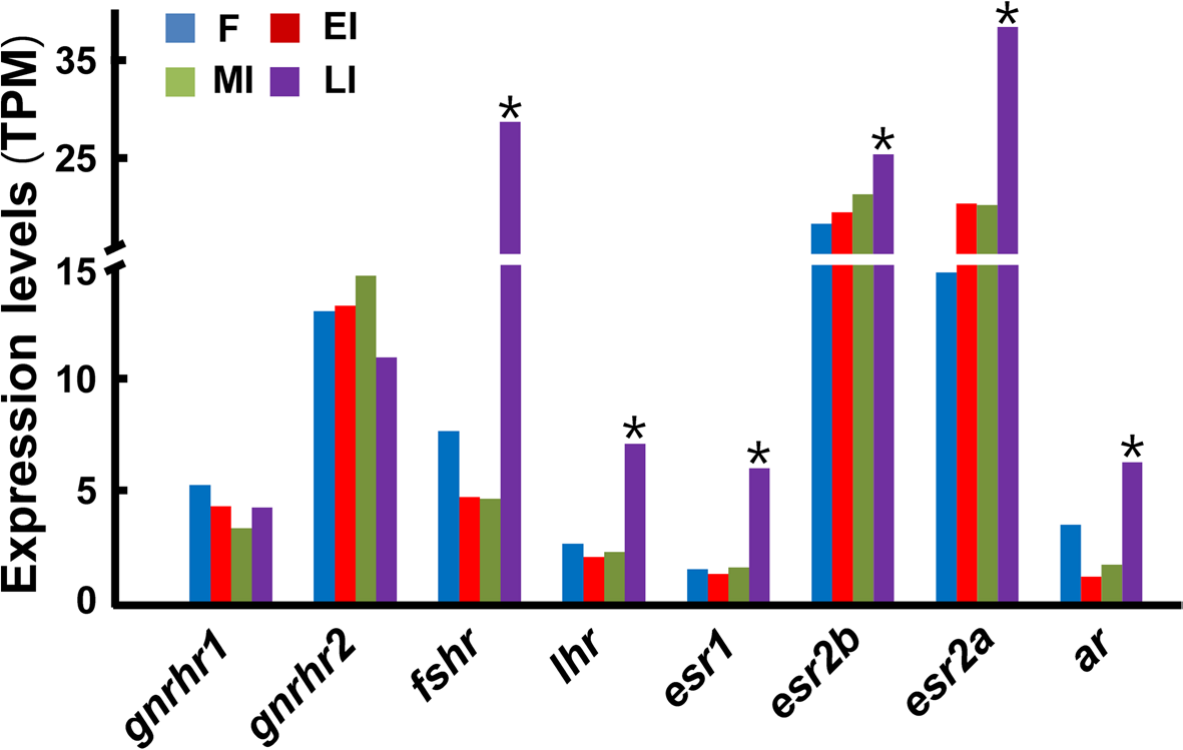
The expression of genes encoding receptors for gonadotropin-releasing hormones, gonadotropins, and sex steroids in gonads of ricefield eels during sex change. Y-axis shows TPM values of genes inferred from the transcriptome data. X-axis shows symbols of genes, and *Entrez* (NCBI) IDs are 109,971,570 (*gnrhr1*), 109,970,856 (*gnrhr2*), 109,961,972 (*lhr*), 109,962,940 (*fshr*), 109,962,316 (*esr2a*), 109,966,606 (*esr2b*), 109,952,736 (*esr1*), and 109,959,413 (*ar*). F: female; EI: early intersexual; MI: mid-intersexual; LI: late intersexual. *up-regulated significantly as compared to that at the previous stage

The expression of *gnrhr2* (gonadotropin-releasing hormone II receptor-like) seemed to be higher than those of *gnrhr1* (gonadotropin-releasing hormone II receptor-like) in gonads at all stages examined, but neither of them showed significant variation during sex change from female to late intersexual stages (Fig. 5). Both gonadotropin receptor genes *lhr* (luteinizing hormone/choriogonadotropin receptor) and *fshr* (follicle stimulating hormone receptor) showed similar expression profiles during sex change, with slightly decreased expression levels at EI and MI as compared to those at F but up-regulated significantly at LI. The expression of two *esr2* genes (*esr2a* for estrogen receptor 2a and *esr2b* for estrogen receptor 2b) seemed to be higher than those of *esr1* (estrogen receptor 1) gene in gonads at all stages examined. The expression of *esr1* was up-regulated significantly at LI while the expression of both *esr2a* and *esr2b* was only slightly increased at LI during sex change. The expression of *ar* (androgen receptor) was slightly decreased at EI and MI but then up-regulated significantly at LI.

### Quantitative real-time PCR (qPCR) validation of gene expression inferred from the transcriptome data

**Fig. 6:**
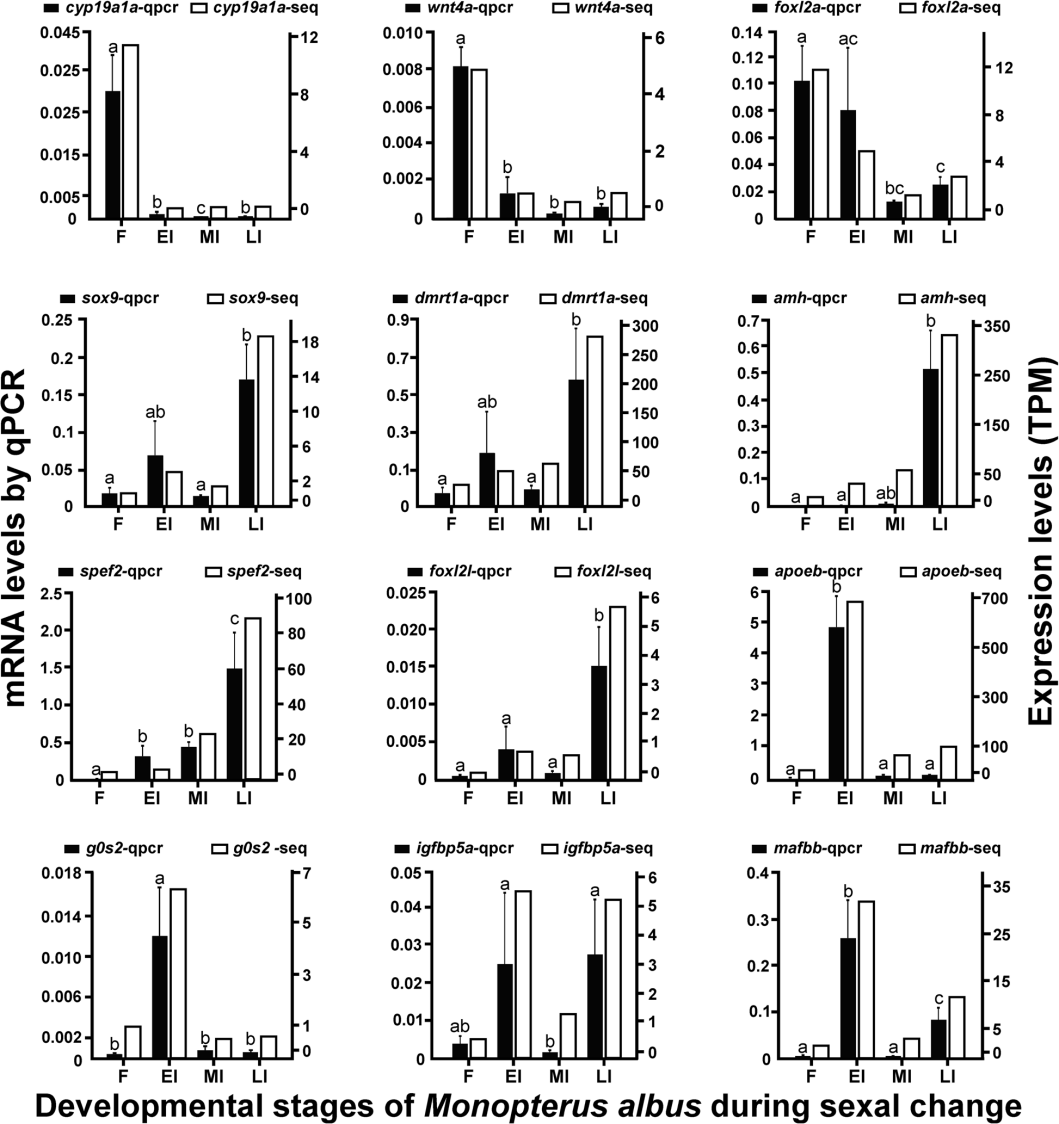
Quantitative real-time PCR (qPCR) validation of the expression of DEGs inferred from the transcriptome data. The left Y-axis and solid bars show mRNA levels by qPCR, and the right Y-axis and empty bars show TPM values inferred from the transcriptome data. The solid bar represents mean ± SEM (*n* = 3). Different letters above the bars indicate significant differences (*p* < 0.05). One-way analysis of variance, followed by Duncan multiple comparison test, was used to estimate significant differences in gene expression across stages except *apoeb* and *amh*, for which Dunnett T3 was used for the multiple comparison test due to the heterogeneity of data. F, female; EI, early intersex; MI, mid-intersex; LI, late intersex

qPCR was performed to validate the expression patterns of 12 transcripts inferred from the transcriptome data, which are presumably associated with sex differentiation (Fig. 6). The female-related genes, including *cyp19a1a*, *wnt4a*, and *foxl2a*, were highly expressed at F, but down-regulated during sex change from MI to LI stages. The male-related genes, including *sox9*, *dmrt1a*, *amh*, and *spef2* (sperm flagellar protein 2), showed low expression levels from F to MI stages but were all highly expressed at LI. Some other genes also showed significant variations during sex change. The expression of *foxl2l* was low from F to MI stages, but increased significantly at LI. The expression of *apoeb* and *g0s2* (G0/G1 switch protein 2) was high at EI but low at the other three stages. *igfbp5a* and *mafbb* were expressed with high abundances at EI and LI but with low abundances at F and MI. The expression profiles of the 12 transcripts as revealed by qPCR analysis were basically similar to those inferred from the transcriptome data (Fig. 6), implying that the analysis of gene expression through RNA-seq data in the present study is reliable.

## Discussion

The ricefield eel, a protogynous hermaphrodite teleost [7, 21], is considered as a model system for vertebrate sexual development [22]. Although many studies have been aimed to identify factors contributing to the sex changing phenomenon of ricefield eels, the underlying mechanisms remain elusive [23]. In the present study, transcriptomic analysis was applied to gonadal samples encompassing the sex changing process from female to late intersexual stages obtained from the same individual ricefield eel through biopsy, which, we believe, is more informative for exploring genes and regulatory pathways related to the sexual change because the genetic backgrounds of the samples are more uniform. The gonadal sexual stages of collected gonadal tissues from individual ricefield eels were carefully verified by histological analysis, which are designated as F (female stage), EI (early intersexual stage), MI (mid-intersexual stage), and LI (late intersexual stage). The assembly of transcriptome data from gonadal cDNA libraries of ricefield eels in the present study resulted in a total of 23,558 genes, of which 20,941 genes were mapped to *Monopterus albus* genome database and 2,617 genes are novel ones. The gene number were comparable to the genome database of *Monopterus albus* updated recently (24,116 genes; https://www.ncbi.nlm.nih.gov/gene/?term=monopterus%20albus), which suggests that the quality of the present transcriptome data is pretty good.

The differential expression of genes along with the gonadal transitional process was analyzed in the following three pairwise comparisons, namely EI vs. F, MI vs. EI, and LI vs. MI. The expression profiles of the selected representative genes across stages inferred from transcriptomic data were similar to those obtained with qPCR, suggesting that transcriptome-based gene expression analysis is essentially reliable. There were about 1.17% of DEGs only identified in the comparisons of EI vs F and MI vs EI. In contrast, about 89.87% of DEGs were identified only in the comparison of LI vs MI, which implies that dramatic developmental changes occurred in gonads from stage MI to LI, and these changes are presumably related to testicular development in ricefield eels.

In protogynous hermaphroditic fish, the decreases of E_2_ and *cyp19a1a* expression were suggested to play essential roles in the sex change from female to male [24–28]. In ricefield eels, similarly, the expression of *cyp19a1a* in gonads was down-regulated from female to male [11, 29]. In support of the above reports, data of the present study indicated that the expression of *cyp19a1a* in gonads was totally blocked at the initial stage of sex change, and remained at very low levels thereafter with TPM below 0.1. These lines of evidence suggest an important role for the down-regulation of *cyp19a1a* in the initiation of sex change in ricefield eels. It has been shown that *foxl2* up-regulates the transcription of *cyp19a1a* of ricefield eels [30] and other teleosts, and plays important roles in the ovarian differentiation [26]. The present transcriptomic data showed that the expression of *foxl2a* was decreased from the female to the mid-intersexual stage. Taken together, these data suggests that the decrease in *foxl2a* expression during sex change may lead to the down-regulation of *cyp19a1a* and contribute to the initiation of sex change in ricefield eels. Dmrt1, a conserved factor essential to testicular development in vertebrates [31], has been demonstrated to inhibit the Ad4BP/SF-1 (Nr5a1a)-activated *cyp19a1a* transcription [32]. Similarly, Dmrt1a was also shown to inhibit Foxl2a-induced activation of ricefield eel *cyp19a1a* promoter (unpublished results). However, whether Dmrt1a contributes to the initiation of sex change through inhibition of *cyp19a1a* or not remains to be elucidated because the expression of *dmrt1a* was increased slightly at the early intersexual stage, and the cellular co-localization of this increased *dmrt1a* mRNA with *cyp19a1a* mRNA needs to be confirmed.

In contrast to *foxl2a*, the expression of the duplicated paralogue *foxl2l* was very low at the female stage, but then increased slightly at the early intersexual stage and significantly at the late intersexual stage. Previously, ricefield eel *foxl2l* was named as *foxl3*, and its expression was shown to be male-biased [33] and barely detectable in ovarian follicles [34]. These lines of evidence support a major role for *foxl2l* in testicular development of ricefield eels [33]. In the bluehead wrasse, a protogynous teleost, the duplicates of *foxl2* also showed contrasting sex-specific expression patterns [35]. Thus Foxl2l of ricefield eel may acquire a novel function after duplication possibly through neofunctionalization during evolution [36].

The Wnt/β-catenin pathway is regarded as a major player in ovarian differentiation in mammals [37], and also possibly involved in the differentiation of the ovary in rainbow trout [38]. The present transcriptomic data showed higher expression of *wnt4a* in the ovary than the gonads of other sexual stages in ricefield eels, which is in line with the major roles of *wnt4* in the ovarian differentiation and maintenance. Fibroblast growth factors (FGF) also play important roles in sex determination and sex differentiation in vertebrates. In mammals, *Fgf9* has been shown to downregulate *Wnt4* to establish the testis pathway in the XY gonad [39]. In teleosts, Fgf homologues other than Fgf9 were identified [40, 41]. In tilapia, *fgf16* and *fgf20b* are highly expressed both in normal XX (genetically female) and sexually changed XY (genetically male) ovaries induced by E_2_, but are low in normal XY testis and AI-induced XX testis, suggesting that *fgf16* and *fgf20b* may play roles in the differentiation of ovaries [42]. Similarly in our present study, *fgf16* was expressed at a higher level at the female stage and down-regulated at EI through LI, implying a major role for *fgf16* in ovarian development of ricefield eels. These lines of evidence suggest that roles of Fgf homologues in gonadal differentiation may be different between mammals and teleosts. Together with the down-regulation of *cyp19a1a*, our present study implied that the inhibition of female pathways is likely a pre-requisite for sexual change in ricefield eels.

In addition to the down-regulated DEGs, the present transcriptomic data identified 603 DEGs that were up-regulated at EI as compared to F. These up-regulated DEGs are presumably closely associated with sex change, and their different expression profiles are suggestive of differential roles in the gonadal transformation process.

Among the 603 upregulated DEGs, the expression of *apoeb*, *csl2*, and *enpp2* was dramatically increased and peaked at EI, but then decreased significantly thereafter. The gene *apoeb* encodes an apolipoprotein E-like protein. It has been shown that in rats, the greatest apolipoprotein E mRNA was expressed in theca cells of atretic follicles, and exogenous apolipoprotein E induced apoptosis of cultured ovarian theca and interstitial cells [43]. The gene *csl2* encodes a L-rhamnose-binding lectin SML-like protein. Lectins are a group of sugar-binding proteins that can stimulate cell proliferation [44]. The gene *enpp2* encodes the ectonucleotide pyrophosphatase/phosphodiesterase 2, which catalyzes the synthesis of lysophosphatidic acid and affects proliferation, cell survival, motility, morphological rearrangements and differentiation [45]. These lines of evidence imply that the upregulation of *apoeb*, *csl2*, and *enpp2* at EI may be predominantly associated with the initiation of sex change in ricefield eels, possibly through apoptosis of ovarian follicular cells, and proliferation and/or differentiation of testicular somatic and germ cells. Their physiological roles and action mechanisms in sex change of ricefield eels are worth further study.

The expression of *mafba*, *clu*, *mafbb*, *igfbp5a*, and *sgk1* showed similar biphasic patterns during sex change of ricefield eels, with two peaks observed at EI and LI, respectively. Interestingly, Mafb is a putative marker for fetal Leydig cell progenitors, and expressed in the testis at the earliest stages of interstitial development in mice [46]. Clusterin (CLU) is a glycoprotein with ubiquitous expression in various tissues and body fluids that acts as an ATP-independent extracellular molecular chaperone. *CLU* overexpression effectively enhanced spermatogenic cell viability and suppressed high glucose-induced apoptosis [47]. The insulin-like growth factor-binding protein-5 (IGFBP-5) is one of the 6 members of the IGFBP family, and is involved in the regulation of cell growth, apoptosis, and other IGF-stimulated signaling pathways [48]. SGK1 (serum and glucocorticoid-inducible kinase 1) is a growth factor-responsive kinase, which has been shown to promote vascular smooth muscle cell proliferation [49]. These lines of evidence indicated that in ricefield eels, *mafba*, *clu*, *mafbb*, *igfbp5a*, and *sgk1* may be associated with the initiation of sexual change and subsequent gonadal development towards testis, possibly through the regulation of follicular atresia and the differentiation and proliferation of male germ and somatic cells including the Leydig cell. Their physiological roles and mechanisms need further study.

The expression of *dzip1*, *flot2*, *emb*, *fsta*, *inhbb*, and *mot1* was continuously increased during sex change, with peaks at LI. The human *DAZ* (Deleted in Azoospermia) genes have been shown to be essential to spermatogenesis [50]. The gene *DZIP1* encodes a DAZ-interacting protein, which is expressed in male germ cells in human, mouse, and rat [51, 52]. *Flot2* gene encodes the protein flotillin 2, which has been shown to be expressed at high levels in male germ cells during mouse spermatogenesis, and be involved in sperm acrosome biogenesis [53]. It is possible that in ricefield eels, both Dzip1 and Flot2 may also play roles in male germ cell development during sex change from female to male.

Follistatin, encoded by *fst*, is an antagonist of activin and can affect the reproductive ability by inhibiting the action of activin [54]. Studies suggested that a reduction in the level of activin signaling may be required for gonocytes to differentiate into spermatogonia in rat testes [55]. Whether the up-regulation of *fsta* during sex change from female to male in ricefield eels is also be associated with the differentiation of spermatogonia is worth further study.

Inhibin, a member of TGF-β superfamily, can act on gonads indirectly through pituitary or directly as a paracrine factor [56]. Inhibin consists of two covalently linked subunits, a common alpha subunit and a beta subunit, the latter of which exists in two forms, A and B (denoted beta A and beta B, respectively). In the male, inhibin B is produced principally by the Sertoli cells in the testis [57]. The expression pattern of *inhbb* in gonads of ricefield eels during sex change probably reflects the differentiation and proliferation of Sertoli cells for the testicular development.

It has been established that developing male germ cells receive an adequate level of energy substrates, preferentially lactate which is produced by the Sertoli cells and transported to germ cells via monocarboxylate transporters (MCTs) including MCT1 [58]. Conditional knockout of *Mct1* (now renamed as *Slc16a1a*) resulted in total absence of spermatozoa and morphological alterations of Sertoli cells [59]. The correct expression, localization, and activity of MCTs depend on interactions with chaperon proteins such as embigin [60]. The parallel increases in the expression of *slc16a1a* and *emb* during sex change from female to the late intersexual stage suggest that both monocarboxylate transporter 1 and embigin may interact to support the spermatogenesis in ricefield eels, possibly through the transfer of lactate from Sertoli cells to germ cells.

Hypothalamic and pituitary hormonal signals were also implicated in the regulation of gonadal sex change in hermaphrodite teleosts, including bluehead wrasse [61, 62], honeycomb grouper [63], and the orange-spotted grouper [64]. In ricefield eels, the precocious sexual change could be induced by the administration of gonadotropin-releasing hormone analogue (sGnRH-A) [10, 65] and ovine-luteinizing hormone (oLH) [66]. Due to the presence of GnRH receptors in the pituitary [67] as well as in the gonad [68], the exogenous GnRH analogue could induce precocious sex change either through indirect actions on the pituitary or direct actions on the gonad. The transcriptomic data of the present study showed that the abundance of either *gnrhr1* or *gnrhr2* did not vary significantly across stages during sex change. In contrast, the abundances of both *lhr* and *fshr* were significantly increased at the late intersexual stage. These lines of evidence favor that GnRH signals most likely induce sex change of ricefield eels indirectly through acting on the pituitary by increasing the synthesis and release of Lh and/or Fsh. As the increases in *lhr* and *fshr* mRNA were significant only at the late intersexual stage rather than the early intersexual stage, these signals presumably mainly contribute to the progression of sex change towards testis from ovotestis but probably not the initiation of sex change.

In contrast to *cyp19a1a*, transcriptomic data showed increased expression of genes encoding estrogen nuclear receptors including *esr1*, *esr2a*, and *esr2b* during natural sex change from female to the late intersexual stage, particularly the *esr1*. Similarly, significant increases in the expression of gonadal estrogen receptors (*esr1*, *esr2a*, and *esr2b*) were observed in the testis of sex-changed juvenile ricefield eels induced by AI treatment [69]. In channel catfish (*Ictalurus punctatus*), *esr1* and *esr2* genes were co-expressed in the mature testis and seemed to be developmentally regulated in spermatocytes [70]. In goldfish (*Carassius auratus*), both ovary and testis had high transcript levels of *esr2a* [71]. Interestingly, exogenous estradiol-17b (E_2_) induced spermatogonial stem cell division in cultured testicular tissue of Japanese eel (*Anguilla japonica*) [72]. These lines of evidence suggest that estrogen and/or estrogen receptors may also play significant roles in the development of testis in ricefield eels as in other teleosts.

## Conclusions

Collectively, data of the present study suggest that initiation of sex change in ricefield eels may require the inhibition of female-related genes, particularly the complete shutdown of *cyp19a1a*, and concurrent activation of some genes like *apoeb* and *enpp2*, etc. Subsequently, progress of sex change from intersexual towards male stages requires up-regulation of male-related genes like *dmrt1* and *amh*, etc. Further studies on the mechanisms of *cyp19a1a* downregulation and the physiological roles and action mechanisms of Apoeb and Enpp2 may help to elucidate the molecular mechanisms underlying the natural sex change of ricefield eels.

## Materials and methods

### Experimental fish and gonadal samples for ontogenic transcriptome analysis

Thirty-six female ricefield eels (body length 29.2 ± 3.4 cm and body weight 20.8 ± 6.7 g) were raised in four indoor concrete ponds (100 × 100 × 90 cm) under natural photoperiods and temperatures from March to April of 2020 in Guangzhou, China, with 9 fish each pond. The ricefield eels were fed with live *Tenebrio molitor* at the daily ration of 5% bodyweight. Serial gonadal samples were obtained through biopsy from each individual fish to trace its gonadal development and sexual change. The biopsy operation was performed by following a previously reported method [19] with minor modifications. Briefly, the fish were first anesthetized with MS-222 (500 mg/L), and then a small incision of about 0.5-1 cm length was made on the right side of ventral body wall ahead of the cloacal orifice of fish. The gonad was identified and separated from the mesentery and the dorsal-intestinal artery with great care to avoid any major injury. A small portion of gonadal tissue was dissected out and cut into two parts. One part was snap frozen in liquid nitrogen for the subsequent extraction of tissue RNA, and the other part was fixed in Bouin solution for histological examination of gonadal stages. After cutting out the gonadal tissue, a unique electronic tag (HT950; Hongteng Barcode Technology Co., Ltd., Guangzhou, China) was implanted into the abdomen for identifying each individual fish. The incision was then sutured and erythromycin ointment was applied to the wound to prevent potential inflammation. The biopsied fish were put in aerated fresh water to recover before returning to the pond. A total of 4 biopsies were made on each individual fish at an interval of about 4 weeks. All procedures and investigations followed the ARRIVE guidelines [73], and were reviewed and approved by the Center for Laboratory Animals of Sun Yat-sen University.

The Bouin-fixed gonadal samples were dehydrated and embedded in paraffin through conventional histological processing. Serial cross sections were cut at 4–5 μm and stained with haematoxylin/eosin. The gonadal sections were examined and photographed with a Nikon DS-Ri2 digital camera on a Nikon E800 microscope (Nikon, Japan). Six of the thirty-six female ricefield eels have undergone sex change during the experiment [18], and from 3 of them serial gonadal samples at the female (F), early intersexual (EI), mid-intersexual (MI), and late intersexual (LI) stages were obtained.

### RNA extraction from gonadal tissues

Total RNA was isolated from serial gonadal samples of the three sex changing fish using Trizol reagent (Invitrogen, USA), and resuspended in RNase-free water. RNA quality was checked with agarose gel electrophoresis and further assessed on an Agilent 2100 Bioanalyser (Agilent Technologies, Palo Alto, CA, USA) before RNA-seq.

### cDNA library construction and sequencing

The construction of cDNA libraries and sequencing were performed essentially as previously described [34]. Briefly, a total amount of 1 μg RNA per sample was used as input material for RNA sequencing. The sequencing library was generated using a NEBNext® Ultra™ RNA Library Prep Kit for Illumina® (#E7530L, NEB, USA) by following the manufacturer’s recommendation. Briefly, mRNA was enriched from total RNA using Oligo (dT) magnetic beads, and fragmented in NEBNext First Strand Synthesis Reaction Buffer (5 ×) by ultrasound. First strand cDNA was synthesized with ProtoScript II Reverse Transcriptase using NEBNext Random Primers. Second-strand cDNAs were synthesized with the Second Strand Synthesis Enzyme Mix in the Second Strand Synthesis Reaction Buffer. Then double-stranded cDNA fragments were purified with 1.8X Agencourt AMPure XP Beads (NEB), end repaired, and ligated to Illumina sequencing adapters. The ligation products were purified with AMPure XP Beads, PCR amplified, and sequenced by Gene Denovo Biotechnology Co. (Guangzhou, China) using Illumina HiSeq 2500 to generate 150 bp paired-end reads.

### Data filtering and annotation

To get high-quality reads, raw data obtained from the sequencing machine were further filtered by fastp (version 0.18.0) to remove reads containing adapters or containing more than 10% of unknown nucleotides (N), and low-quality reads containing more than 50% of low-quality bases (Q-value ≤ 20). At the same time, Q20, Q30, GC content, and the sequence duplication levels of the high-quality data were calculated. All the downstream analysis was based on data of high quality. Short reads alignment tool Bowtie2 [74] (version 2.2.8) was used to map reads to the ribosome RNA (rRNA) database of ricefield eels, and the rRNA mapped reads were removed for gene abundance calculation. An index of the reference genome was built, and paired-end high-quality reads were mapped to the ricefield eel *M. albus* genome database (https://www.Ncbi.nlm.nih.gov/genome/24053?genome_assembly_id=302095), using HISAT2.2.4 [75] with “RNA—strandness RF” and other parameters set as default. There were some transcripts that were aligned to the unannotated regions in the genome database of ricefield eel, and these transcripts were called novel genes in the present study. Then the novel genes were further aligned by using BLASTx program (http://www.ncbi.nlm.nih.gov/BLAST/) with an E-value threshold of 1e-5 to the NCBI non-redundant protein (Nr) database (http://www.ncbi.nlm.nih.gov), the Swiss-Prot protein database (http://www.expasy.ch/sprot), the Kyoto Encyclopedia of Genes and Genomes (KEGG) database (http://www.genome.jp/kegg), and the Gene Ontology database (http://www.geneontology.org/). Protein functional annotations could then be obtained for the novel genes according to the best alignment results.

### Gene expression analysis

The mapped reads of each sample were assembled by using StringTie v1.3.1 [76, 77] in a reference-based approach. For each transcription region, a TPM (transcripts per million) value [78] was calculated to quantify its expression abundance and variations using String Tie software.

A differential gene expression analysis between pairs of samples (F vs. EI, EI vs. MI, MI vs. LI) of three fish was performed by using GLM with negative binomial distribution under paired statistical model in edgeR [79]. The genes/transcripts with the parameter of false discovery rate (FDR) below 0.05 and absolute fold change ≥ 2 were considered to be differentially expressed. All differentially expressed genes (DEGs) were mapped to GO terms in the Gene Ontology database (http://www.geneontology.org/), and gene numbers were calculated for every term. The Kyoto Encyclopedia of Genes and Genomes (KEGG) database was used to interpret the biological functions of differentially expressed genes. The significantly enriched GO terms and KEGG pathways in differentially expressed genes compared to the genome background were defined by hypergeometric test. The calculated p-value of both enrichment analyses was gone through FDR correction, taking FDR (Q-value) ≤ 0.05 as a threshold. GO terms or KEGG pathways meeting this criterion were defined as significantly enriched in DEGs.

### Validation of differentially expressed genes from transcriptomes with real-time quantitative PCR

The relative mRNA levels of 12 differentially expressed genes identified from transcriptomes were examined by real-time quantitative PCR (qPCR) to verify their expression profiles in ricefield eel gonads during sexual change from female to late intersexual stages. The main purpose of analyzing the expression of these 12 genes by qPCR was to test if their expression profiles parallel with those inferred from RNA-seq data. Three biological replicates were employed for each stage and processed similarly as reported [34]. Briefly, total RNA (1 μg) from each sample was first treated with RNase-free DNase I and then reverse transcribed with random primers by using the RevertAid H Minus First Strand cDNA Synthesis Kit (Fermentas, Vilnius, Lithuania) according to the manufacturer’s instruction. The qPCR reaction was performed on the Roche Light Cycler 480 in a reaction volume of 10 μl containing 0.3 µM of each primer, 1 µL of cDNA template, and 5 µL of 2 × Bestar® SYBR Green qPCR Master Mix (DBI® Bioscience, Germany). The oligonucleotide primers (Table 1) were designed to span exons when possible. The cycling parameters were: 95 °C for 5 min, followed by 40 cycles of amplification at 95 °C for 10 s, 58 °C for 15 s and 72 °C for 20 s. The specificity of qPCR amplification was confirmed by melt-curve analysis, agarose gel electrophoresis, and sequencing of PCR products.

**Table 1:** Sequences of primers used for real-time quantitative PCR analysis

Gene ID	Genes	Forward primer (5’-3’)	Reverse primer (5’-3’)
109,972,432	*actb*	GAAGACGAAATCGCCGCACTC	GCCCATACCCACCATCACTCC
109,966,231	*gadph*	CTGAAGGGCATTCTGGGA	GCTGTAGCCGAACTCATTGTC
109,957,597	*hprt1*	GACCGCTCCATCCCAAT	CGCCACCGATTACTTTGA
109,963,333	*cyp19a1a*	GCCACTTTTGTCATATGTGAGATTC	CCACAGAGCTACGTTGTTGTTAAAT
109,971,989	*wnt4*	AGGCCTCATCCAGAGACAGGTCCAG	TGGCTGCTGAGATGGCATACACAAA
109,951,921	*foxl2*	GTTCAGACCTCCACCTACGCACTTC	AGTCCAGTGAACGTGAAGGGGTTG
109,954,649	*apoeb*	GCAGAACTCCGATAATGTCC	TCTTCAGCATGGAAGAGAGG
109,955,915	*mafbb*	CGGCATCAAGAAGGAGG	GGGTGTCAGGCCAAAAG
109,968,774	*igfbp5a*	TATGCAGGAACGAGAAGTTG	CTGTCTCTTACGGTCTAGGG
109,971,534	*foxl3*	GGCGGCGGAAGAGAGTTAGGAGACC	CCCCGTAGGGAGGGTGGTTGAAGTG
109,973,075	*amh,*	CATCAGAGGGCAACATTTAC	CAAACCCTGTCCTTCCTT
109,973,215	*sox9*	GTGTCCCAGGTATTGAAAGG	AACGGACGCTTTTCTACTTC
109,956,543	*spef2*	TCATTCAGGTTGGGTCC	TGTGTGAAATCTGTGCCA
109,951,421	*dmrt1a*	TCCACCACCTGTGTGCCACCCATCT	TGAAGTTGACTGTCTCGCTGCCGCC
MSTRG.21213	*g0s2*	ATGAAGAAGAAGCAGGTGTTG	GCATGTAAGCGGTTGGC

The quantification of the mRNA expression level was conducted as previously described [80], using a standard curve with tenfold serial dilutions of the plasmid containing the corresponding cDNA fragment which ranges from 10^1^ to 10^9^ copies. The correlation coefficients were not less than 0.98, and the PCR efficiencies ranged from 1.9 to 2.0. To minimize variation due to the differences in RNA loading, the geometric mean expression levels of *actb*, *gapdh*, and *hprt1* were used to normalize the expression levels of the target genes. One-way analysis of variance, followed by the Duncan multiple comparison test, was used to estimate significant differences (*p* < 0.05) in gene expression across sexual stages except *apoeb* and *amh*, for which Dunnett T3 was used for the multiple comparison test due to the heterogeneity of data. 

## Supplementary Information

**Additional file 1:** **Figure S1.** Representative total RNA extracted from gonadal tissues of ricefield eels at stages of female (F), early intersex (EI), mid-intersex (MI), and late intersex (LI). **Figure S2.** Gene ontology classifications of DEGs in the transcriptome data of ricefield eel gonads during sex change. A: EI vs. F. B: MI vs. EI. C: LI vs MI. X-axis shows the GO term. Y-axis shows the number of DEGs. F: female; EI: early intersex; MI: mid-intersex; LI: late intersex.

**Additional file 2:** **Table S1.** Quality control parameters of the sequencing data. **TableS2.** Summary of high-quality reads mapped to the ribosome database. **Table S3.** Overview of mapping status for gonadal transcriptomes during sexual change of ricefield eel. **Table S4.** Number of reference genes and new genes in sequencing data. **Table S5.** Annotation of novel genes.**Table S6.** GO Enrichment Analysis of DEGs between EI vs F. **Table S7.** GO Enrichment Analysis of DEGs between MI vs EI. **Table S8.** GO Enrichment Analysis of DEGs between LI vs MI. **Table S9.** KEGG enrichment analysis of DEGs between EI vs F. **Table S10.** KEGG enrichment analysis of DEGs between MI vs EI. **Table S11**. KEGG enrichment analysis of DEGs between LI vs MI. **Table S12.** The expression of down-regulated DEGs for EI vs F during sex change. **Table S13.** The expression of up-regulated for DEGs for EI vs F during sex change.

## Data Availability

The Illumina HiSeq 2500 was used to generate transcriptomic data on gonadal samples. All data were deposited at the NCBI under Bioproject PRJNA764057 and are publicly available (https://www.ncbi.nlm.nih.gov/search/all/?term = PRJNA764057).
