# Supplementary material for: The ontogenic gonadal transcriptomes provide insights into sex change in the ricefield eel Monopterus albus
Source: BMC Zool. 2022 Nov 23;7:56. doi: 10.1186/s40850-022-00155-4 (PMC10127409; doi:10.1186/s40850-022-00155-4)
Supplement: Supplementary file 1 — Additional file 1: Figure S1. Representative total RNA extracted from gonadal tissues of ricefield eels at stages of female (F), early intersex (EI), mid-intersex (MI), and late intersex (LI). Figure S2. Gene ontology classifications of DEGs in the transcriptome data of ricefield eel gonads during sex change. A: EI vs. F. B: MI vs. EI. C: LI vs MI. X-axis shows the GO term. Y-axis shows the number of DEGs. F: female; EI: early intersex; MI: mid-intersex; LI: late intersex. [file 40850_2022_155_MOESM1_ESM.pdf]

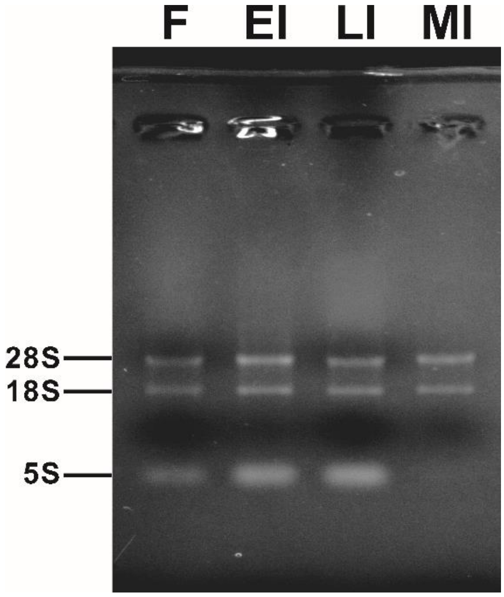

Figure S1. Representative total RNA extracted from gonadal tissues of ricefield eels at stages of female (F), early intersex (EI), mid-intersex (MI), and late intersex (LI).

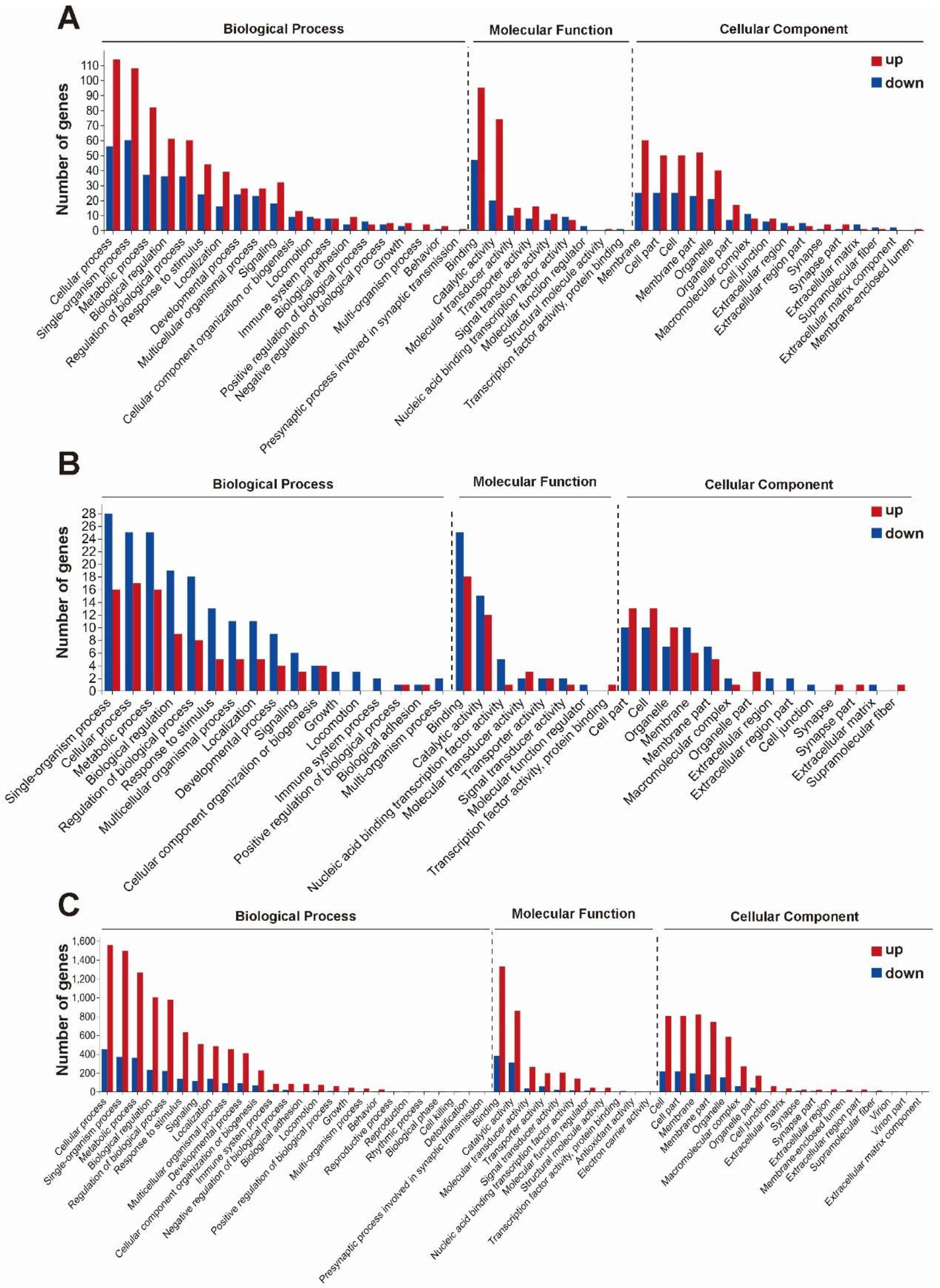

Figure S2. Gene ontology classifications of DEGs in the transcriptome data of ricefield eel gonads during sex change. A: EI vs. F. B: MI vs. EI. C: LI vs MI. X-axis shows the GO term. Y-axis shows the number of DEGs. F: female; EI: early intersex; MI: mid-intersex; LI: late intersex.
